# Supplementary figures and images for: Distributional dynamics of a vulnerable species in response to past and future climate change: a window for conservation prospects
Source: PeerJ. 2018 Jan 16;6:e4287. doi: 10.7717/peerj.4287 (PMC5774295; doi:10.7717/peerj.4287)

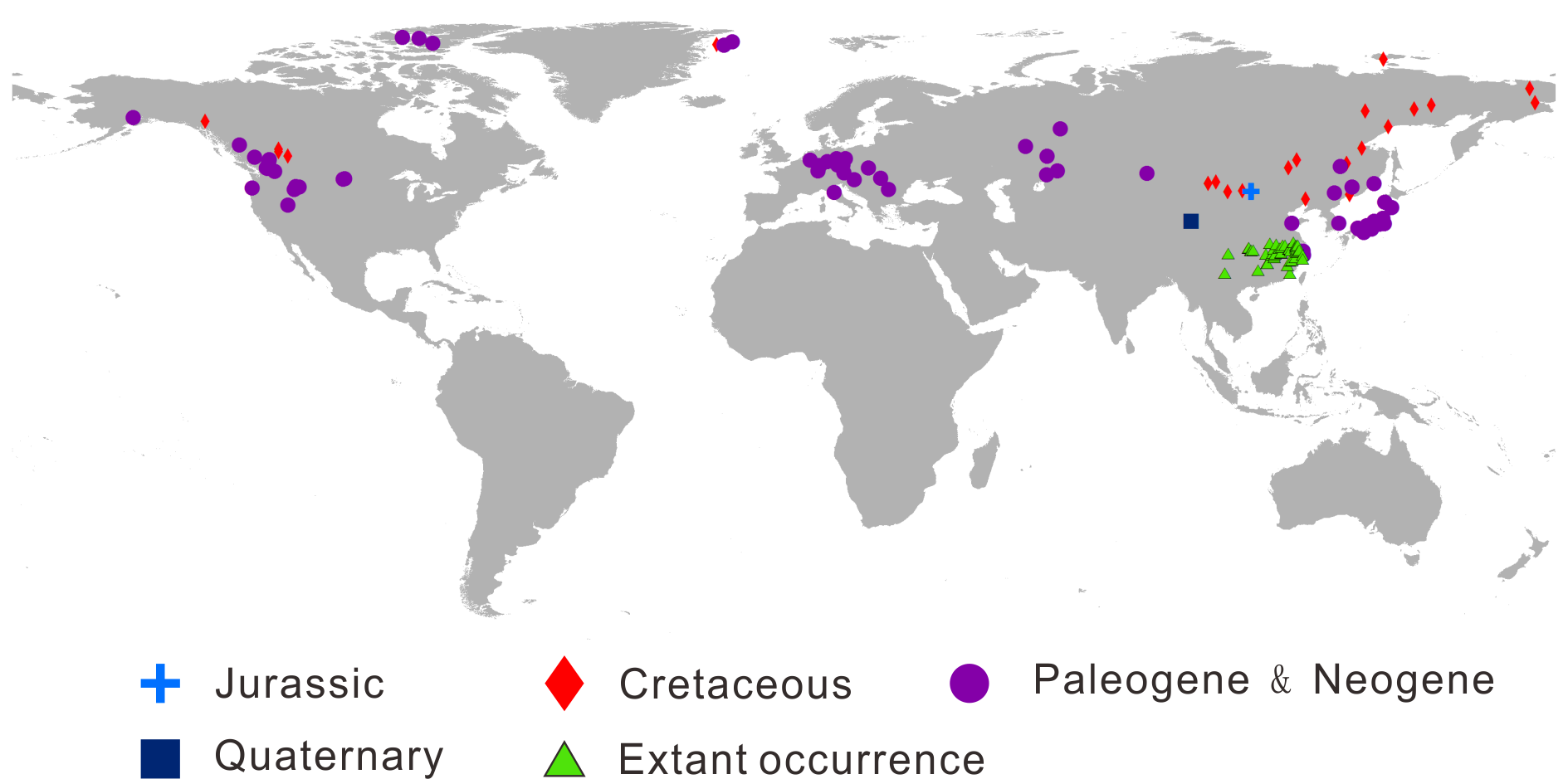

Supplement: Figure S1 — The spatial data was freely downloaded from http://www.diva-gis.org/Data, the base map was generated by ArcGIS v.9.3 (http://www.esri.com/software/arcgis/arcgis-for-desktop). [file peerj-06-4287-s001.png]

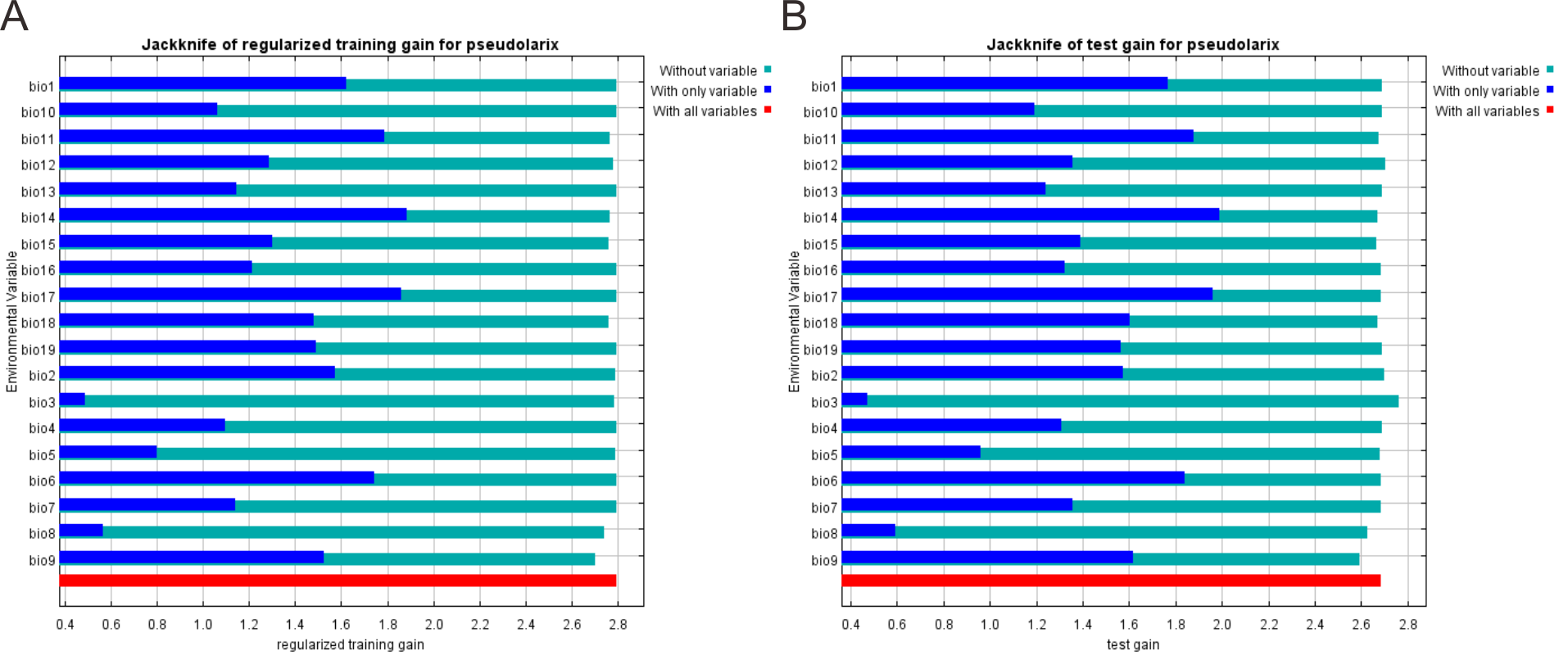

Supplement: Figure S2 — The Jackknife test for evaluating the contribution of all 19 climate variables from the WorldClim dataset for model training (A) and testing (B). Bio1: annual mean temperature, Bio2: mean diurnal range (mean of monthly (max temp - min temp)), Bio3: isothermality (Bio2 / Bio7) (* 100), Bio4: temperature seasonality (standard deviation *100), Bio5: max temperature of warmest month, Bio6: min temperature of coldest month, Bio7: temperature annual range (Bio5–Bio6), Bio8: mean temperature of wettest quarter, Bio9: mean temperature of driest quarter, Bio10: mean temperature of warmest quarter, Bio11: mean temperature of coldest quarter, Bio12: annual precipitation, Bio13: precipitation of wettest month, Bio14: precipitation of driest month, Bio15: precipitation seasonality (coefficient of variation), Bio16: precipitation of wettest quarter, Bio17: precipitation of driest quarter, Bio18: precipitation of warmest quarter, Bio19: precipitation of coldest quarter. [file peerj-06-4287-s002.png]

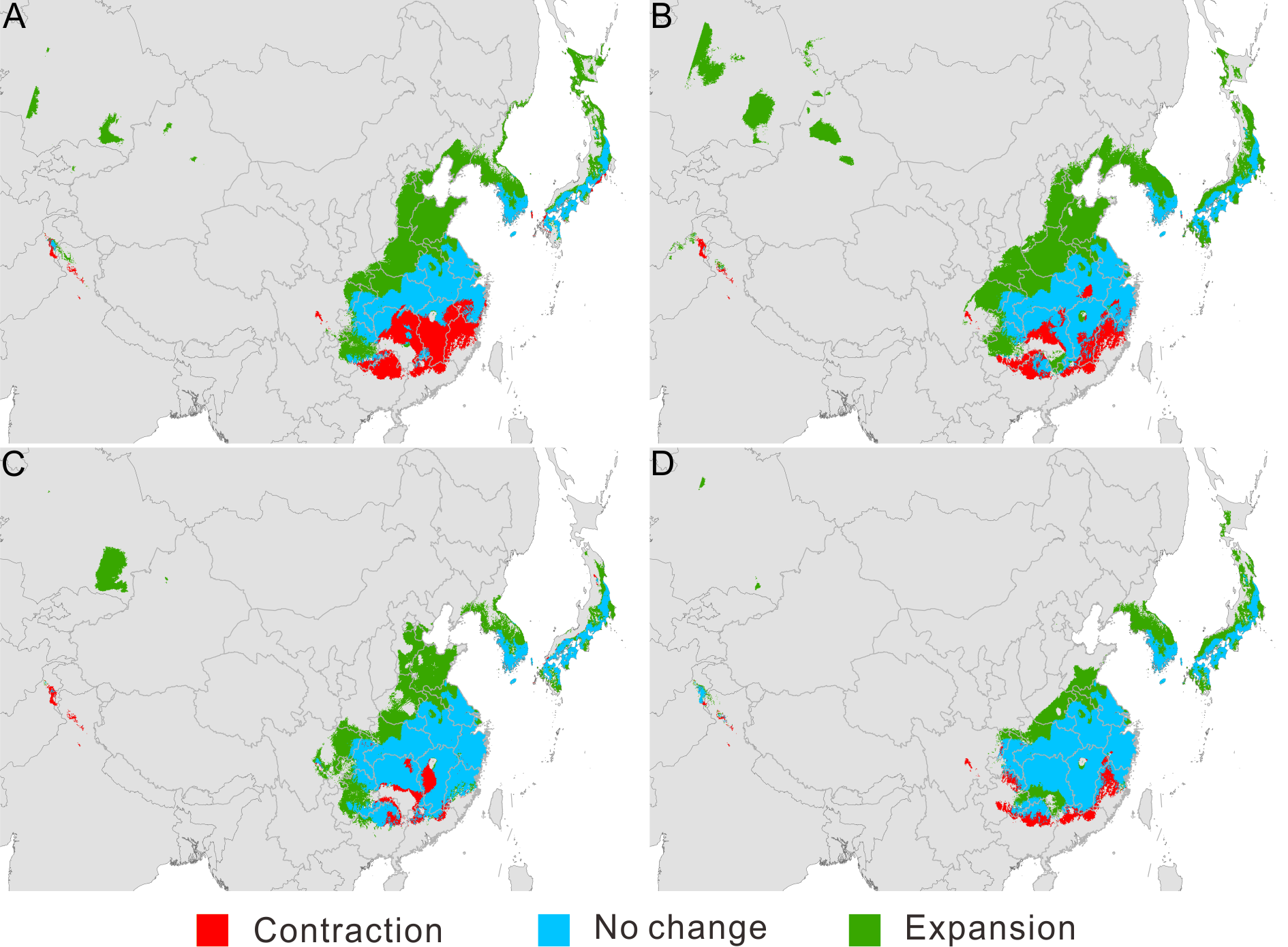

Supplement: Figure S3 — Four types of predictions of climate suitability for Pseudolarix amabilis in the future (2080) using climate layers generated by the following: UK Meteorological Office (UKMO) Hadley Centre Coupled Model, version 3 (HadCM3), under SRES A1B (A); Met Office Hadley Centre (MOHC) Hadley Centre Global Environmental Model, version 2 (HadGEM2-ES (Earth System)), under RCP 8.5 (B); Canadian Centre for Climate Modelling and Analysis (CCCma) third-generation Coupled Global Climate Model with T63 spectral resolution (CGCM3.1-T63) under SRES A1B (C); and Meteorological Research Institute (MRI) Coupled Global Climate Model, version 3 (CGCM3), under RCP 8.5 (D). The spatial data was freely downloaded from http://www.diva-gis.org/Data, the base map was generated by ArcGIS v.9.3 (http://www.esri.com/software/arcgis/arcgis-for-desktop). [file peerj-06-4287-s003.png]

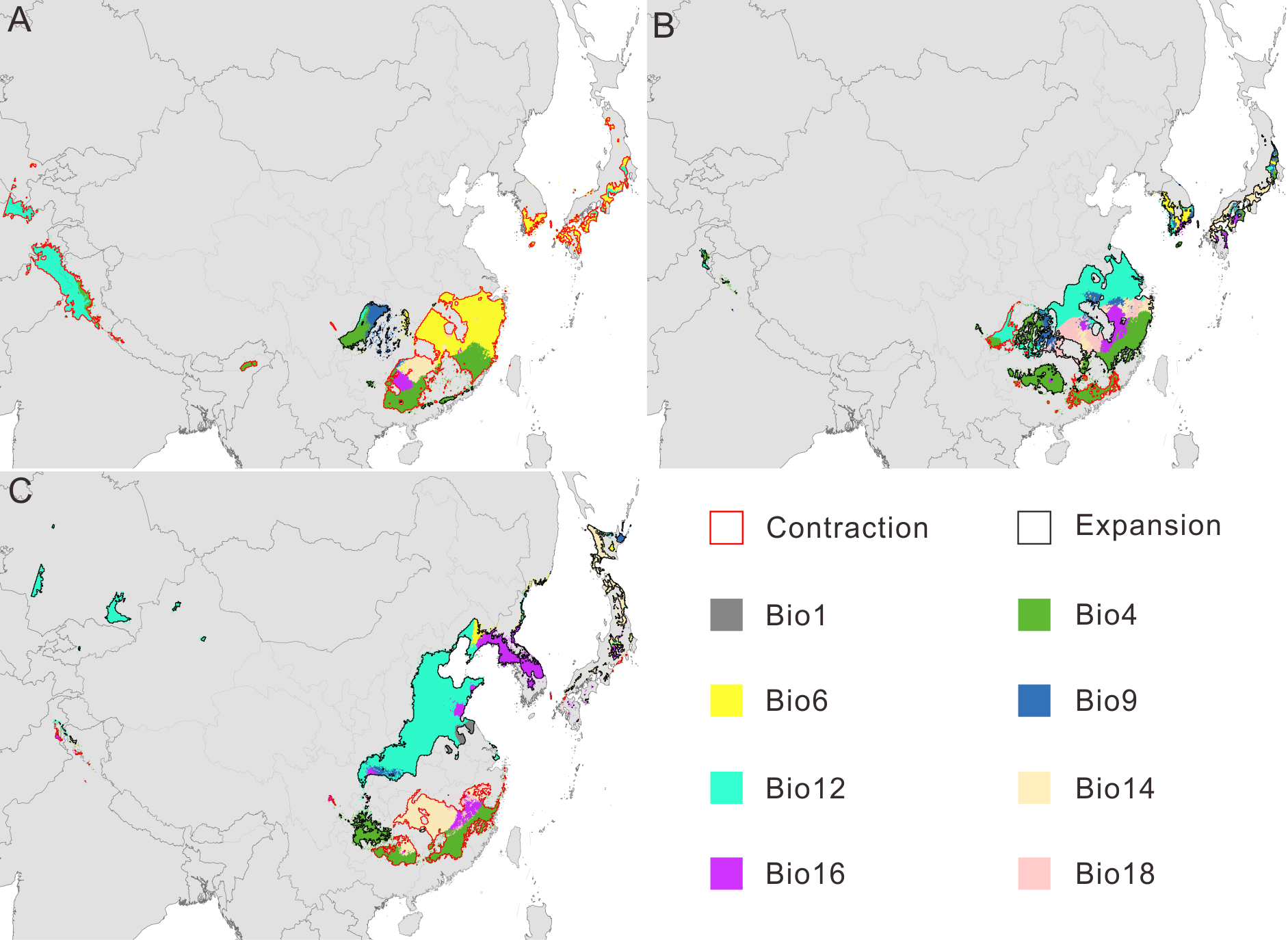

Supplement: Figure S4 — All the limiting climatic factors responsible for the contraction and expansion of distribution of Pseudolarix amabilis over climate transitions: from the LIG to the LGM (A), from the LGM to the present (B), and from the present to 2080 (C). Bio1: annual mean temperature, Bio4: temperature seasonality (standard deviation *100), Bio6: min temperature of coldest month, Bio9: mean temperature of driest quarter, Bio12: annual precipitation, Bio14: precipitation of driest month, Bio16: precipitation of wettest quarter, Bio18: precipitation of warmest quarter. The spatial data was freely downloaded from http://www.diva-gis.org/Data, the base map was generated by ArcGIS v.9.3 (http://www.esri.com/software/arcgis/arcgis-for-desktop). [file peerj-06-4287-s004.png]

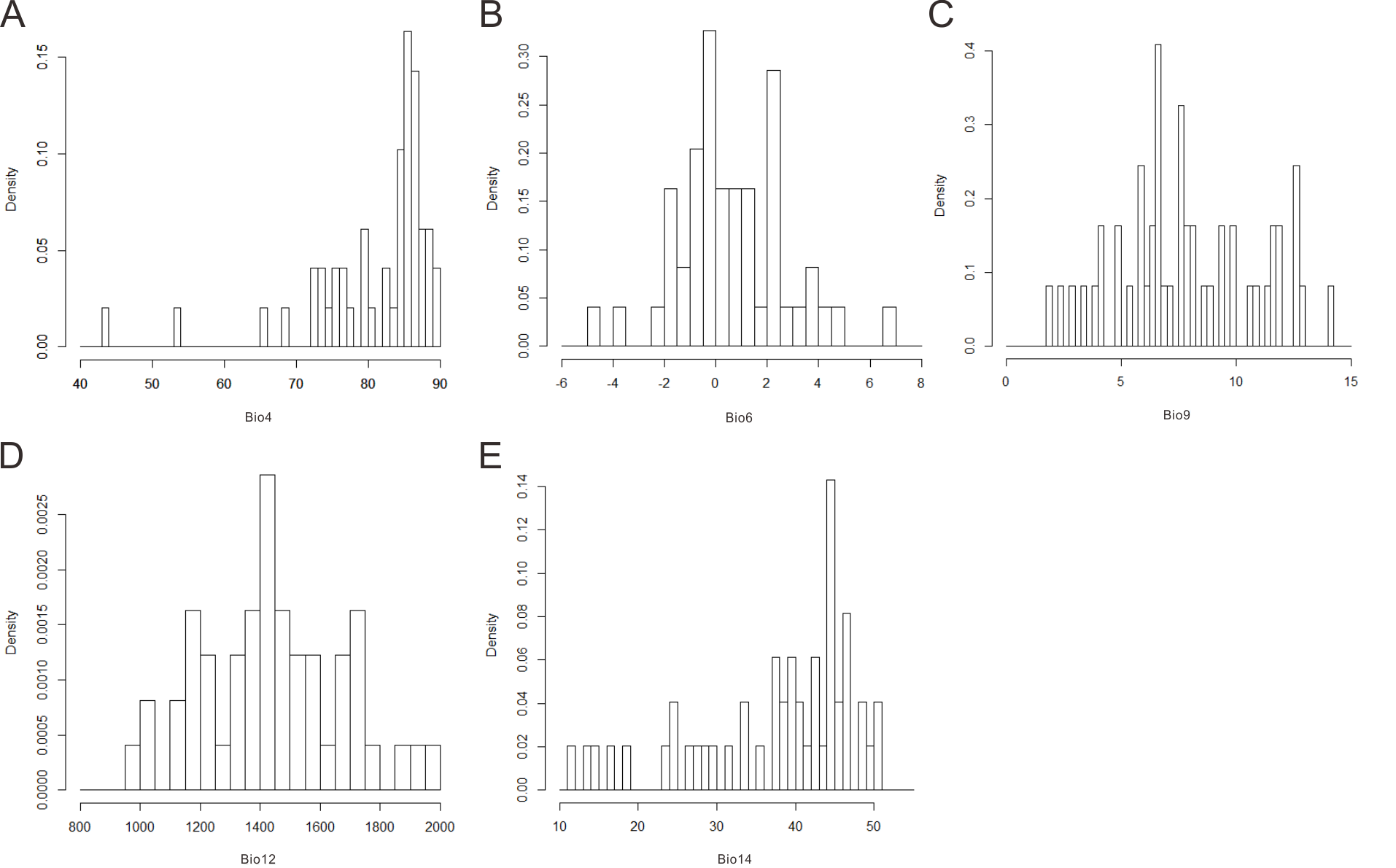

Supplement: Figure S5 — Bio4: temperature seasonality (A), Bio6: min temperature of coldest month (°C) (B), Bio9: mean temperature of driest quarter (°C) (C), Bio12: annual precipitation (mm) (D), Bio14: precipitation of driest month (mm) (E). [file peerj-06-4287-s005.png]
