# Supplementary material for: Distributional dynamics of a vulnerable species in response to past and future climate change: a window for conservation prospects
Source: PeerJ. 2018 Jan 16;6:e4287. doi: 10.7717/peerj.4287 (PMC5774295; doi:10.7717/peerj.4287)
Supplement: Table S1 [file peerj-06-4287-s006.docx]

|  | Bio1 | Bio2 | Bio3 | Bio4 | Bio5 | Bio6 | Bio7 | Bio8 | Bio9 | Bio10 | Bio11 | Bio12 | Bio13 | Bio14 | Bio15 | Bio16 | Bio17 | Bio18 | Bio19 |
| --- | --- | --- | --- | --- | --- | --- | --- | --- | --- | --- | --- | --- | --- | --- | --- | --- | --- | --- | --- |
| Bio1 | 1 |  |  |  |  |  |  |  |  |  |  |  |  |  |  |  |  |  |  |
| Bio2 | 0.246 | 1 |  |  |  |  |  |  |  |  |  |  |  |  |  |  |  |  |  |
| Bio3 | 0.174 | 0.65 | 1 |  |  |  |  |  |  |  |  |  |  |  |  |  |  |  |  |
| Bio4 | -0.068 | -0.047 | -0.761 | 1 |  |  |  |  |  |  |  |  |  |  |  |  |  |  |  |
| Bio5 | 0.788 | 0.214 | -0.302 | 0.532 | 1 |  |  |  |  |  |  |  |  |  |  |  |  |  |  |
| Bio6 | 0.835 | -0.021 | 0.346 | -0.531 | 0.386 | 1 |  |  |  |  |  |  |  |  |  |  |  |  |  |
| Bio7 | 0.017 | 0.219 | -0.582 | **0.957** | 0.604 | -0.503 | 1 |  |  |  |  |  |  |  |  |  |  |  |  |
| Bio8 | 0.323 | 0.018 | -0.198 | 0.369 | 0.442 | 0.117 | 0.313 | 1 |  |  |  |  |  |  |  |  |  |  |  |
| Bio9 | 0.604 | 0.129 | 0.213 | -0.201 | 0.389 | 0.584 | -0.141 | 0.107 | 1 |  |  |  |  |  |  |  |  |  |  |
| Bio10 | 0.799 | 0.168 | -0.318 | 0.544 | **0.984** | 0.388 | 0.586 | 0.505 | 0.39 | 1 |  |  |  |  |  |  |  |  |  |
| Bio11 | **0.851** | 0.206 | 0.533 | -0.579 | 0.362 | **0.965** | -0.494 | 0.086 | 0.619 | 0.368 | 1 |  |  |  |  |  |  |  |  |
| Bio12 | -0.003 | -0.285 | -0.123 | -0.178 | -0.093 | 0.14 | -0.208 | -0.382 | 0.014 | -0.127 | 0.084 | 1 |  |  |  |  |  |  |  |
| Bio13 | 0.063 | -0.015 | 0.183 | -0.37 | -0.163 | 0.216 | -0.339 | -0.523 | 0.131 | -0.189 | 0.229 | 0.784 | 1 |  |  |  |  |  |  |
| Bio14 | 0.063 | -0.168 | -0.393 | 0.368 | 0.249 | -0.116 | 0.333 | -0.127 | 0.27 | 0.257 | -0.131 | 0.648 | 0.367 | 1 |  |  |  |  |  |
| Bio15 | 0.064 | 0.311 | 0.717 | -0.718 | -0.36 | 0.335 | -0.626 | -0.218 | 0.059 | -0.377 | 0.411 | -0.177 | 0.323 | -0.67 | 1 |  |  |  |  |
| Bio16 | 0.063 | 0.024 | 0.255 | -0.439 | -0.187 | 0.247 | -0.389 | -0.535 | 0.082 | -0.23 | 0.263 | 0.83 | **0.956** | 0.347 | 0.332 | 1 |  |  |  |
| Bio17 | 0.085 | -0.15 | -0.397 | 0.389 | 0.273 | -0.118 | 0.357 | -0.122 | 0.172 | 0.286 | -0.127 | 0.667 | 0.356 | **0.977** | -0.695 | 0.349 | 1 |  |  |
| Bio18 | -0.31 | -0.265 | 0.11 | -0.391 | -0.489 | -0.007 | -0.452 | -0.207 | -0.254 | -0.506 | -0.058 | 0.658 | 0.664 | 0.096 | 0.323 | 0.691 | 0.113 | 1 |  |
| Bio19 | 0.16 | -0.048 | -0.255 | 0.246 | 0.266 | -0.007 | 0.256 | -0.197 | 0.388 | 0.265 | 0.011 | 0.684 | 0.484 | **0.957** | -0.551 | 0.462 | **0.94** | 0.08 | 1 |

Notes: Bold fonts indiate the correlation coefficients that are greater than 0.85. Bio1: annual mean temperature, Bio2: mean diurnal range (mean of monthly (max temp - min temp)), Bio3: isothermality (Bio2 / Bio7) (* 100), Bio4: temperature seasonality (standard deviation *100), Bio5: max temperature of warmest month, Bio6: min temperature of coldest month, Bio7: temperature annual range (Bio5 - Bio6), Bio8: mean temperature of wettest quarter, Bio9: mean temperature of driest quarter, Bio10: mean temperature of warmest quarter, Bio11: mean temperature of coldest quarter, Bio12: annual precipitation, Bio13: precipitation of wettest month, Bio14: precipitation of driest month, Bio15: precipitation seasonality (coefficient of variation), Bio16: precipitation of wettest quarter, Bio17: precipitation of driest quarter, Bio18: precipitation of warmest quarter, Bio19: precipitation of coldest quarter.
