# Supplementary material for: Distributional dynamics of a vulnerable species in response to past and future climate change: a window for conservation prospects
Source: PeerJ. 2018 Jan 16;6:e4287. doi: 10.7717/peerj.4287 (PMC5774295; doi:10.7717/peerj.4287)
Supplement: Table S2 [file peerj-06-4287-s007.docx]

| Species | Longitude(°N) | Latitude(°E) | Species | Longitude(°N) | Latitude(°E) |
| --- | --- | --- | --- | --- | --- |
| *P. amabilis* | 102.83 | 24.88 | *P. amabilis* | 116.08 | 31 |
| *P. amabilis* | 118.09 | 24.88 | *P. amabilis* | 116.32 | 31.4 |
| *P. amabilis* | 103.7 | 29.47 | *P. amabilis* | 116.95 | 31.46 |
| *P. amabilis* | 108.31 | 30.71 | *P. amabilis* | 117.47 | 26.73 |
| *P. amabilis* | 108.41 | 30.81 | *P. amabilis* | 117.83 | 30.5 |
| *P. amabilis* | 108.94 | 30.29 | *P. amabilis* | 117.85 | 30.64 |
| *P. amabilis* | 109 | 30.14 | *P. amabilis* | 117.93 | 30.62 |
| *P. amabilis* | 109.49 | 30.27 | *P. amabilis* | 118.04 | 27.76 |
| *P. amabilis* | 110.59 | 25.6 | *P. amabilis* | 118.17 | 30.15 |
| *P. amabilis* | 112.41 | 29.37 | *P. amabilis* | 118.54 | 27.92 |
| *P. amabilis* | 112.73 | 27.24 | *P. amabilis* | 118.8 | 32.06 |
| *P. amabilis* | 112.74 | 27.23 | *P. amabilis* | 119.28 | 28.59 |
| *P. amabilis* | 112.81 | 27.22 | *P. amabilis* | 119.37 | 30.2 |
| *P. amabilis* | 113.31 | 31.86 | *P. amabilis* | 119.38 | 31.42 |
| *P. amabilis* | 113.82 | 29.25 | *P. amabilis* | 119.42 | 30.89 |
| *P. amabilis* | 114.04 | 29.56 | *P. amabilis* | 119.49 | 31.27 |
| *P. amabilis* | 114.31 | 30.59 | *P. amabilis* | 119.51 | 31.18 |
| *P. amabilis* | 114.37 | 28.52 | *P. amabilis* | 119.6 | 30.52 |
| *P. amabilis* | 114.41 | 29.01 | *P. amabilis* | 119.69 | 29.79 |
| *P. amabilis* | 114.57 | 29.36 | *P. amabilis* | 119.82 | 31.38 |
| *P. amabilis* | 114.88 | 31.64 | *P. amabilis* | 119.96 | 30.05 |
| *P. amabilis* | 115.68 | 30.74 | *P. amabilis* | 120.17 | 30.27 |
| *P. amabilis* | 115.99 | 29.67 | *P. amabilis* | 120.98 | 29.14 |
| *P. amabilis* | 116 | 29.61 | *P. amabilis* | 121.02 | 28.25 |
| *P. amabilis* | 116 | 29.71 |  |  |  |
